# Aging Reduces the Functional Brain Networks Strength—a Resting State fMRI Study of Healthy Mouse Brain

**DOI:** 10.3389/fnagi.2019.00277

**Published:** 2019-10-11

**Authors:** Ander Egimendia, Anuka Minassian, Michael Diedenhofen, Dirk Wiedermann, Pedro Ramos-Cabrer, Mathias Hoehn

**Affiliations:** ^1^Magnetic Resonance Imaging Laboratory, CIC biomaGUNE, Donostia-San Sebastián, Spain; ^2^Multiple Sclerosis Unit, Biodonostia Health Institute, Donostia-San Sebastián, Spain; ^3^In-vivo-NMR Laboratory, Max Planck Institute for Metabolism Research, Cologne, Germany; ^4^Ikerbasque—Basque Foundation for Science, Bilbao, Spain

**Keywords:** functional neuronal networks, aging, dependence on aging, mice, resting state fMRI, seed correlation analysis, brain connectivity

## Abstract

Resting-state functional magnetic resonance imaging (rsfMRI) is increasingly used to unravel the functional neuronal networks in health and disease. In particular, this technique of simultaneously probing the whole brain has found high interest in monitoring brain wide effects of cerebral disease and in evaluating therapeutic strategies. Such studies, applied in preclinical experimental mouse models, often require long-term observations. In particular during regeneration studies, easily several months of continuous monitoring are required to detect functional improvements. These long periods of following the functional deficits during disease evolution as well as the functional recoveries during therapeutic interventions represent a substantial fraction of the life span of the experimental animals. We have therefore aimed to decipher the role of healthy aging alone for changes in functional neuronal networks in mice, from developmental adolescence *via* adulthood to progressing aging. For this purpose, four different groups of C57Bl6 mice of varying age between 2 and 13 months were studied twice with 4 weeks separation using resting state fMRI at 9.4T. Dedicated data analysis including both Independent Component Analysis (ICA) followed by seed-based connectivity matrix compilation resulted in an inverse U-shape curve of functional connectivity (FC) strength in both the sensorimotor and default mode network (DMN). This inverse U-shape pattern presented a distinct maximum of FC strength at 8–9 months of age, followed by a continuous decrease during later aging phases. At progressed aging at 12–13 months, the reduction of connectivity strength varied between 25% and 70% with most connectivities showing a reduction in strength by approximately 50%. We recommend that these substantial age-dependent changes in FC strength must be considered in future longitudinal studies to discriminate focused disease-based functional deficits and therapy-related functional improvements from underlying independent age effects.

## Introduction

Mouse models have become the cornerstone of research for neurodegenerative diseases such as multiple sclerosis (Binnewijzend et al., 2012; Ransohoff, 2012), Alzheimer’s disease (Busche et al., 2008), Parkinson’s disease (Antony et al., 2011) or amyotrophic lateral sclerosis (Lutz, 2018). This has been achieved due to the opportunities that mice offer to be genetically manipulated along with the continuous discovery of gene mutations related to many neurodegenerative pathologies (Huang et al., 2011).

In this context, functional magnetic resonance imaging (fMRI) has become a crucial tool for the study of functional deficits in brain diseases and of functional improvements due to therapeutic intervention, respectively (Ramos-Cabrer et al., 2010). Resting-state fMRI (rsfMRI) is non-invasive and measures at high spatial resolution blood oxygen level dependent (BOLD) patterns at low frequencies in the absence of external stimuli (Biswal et al., 1995). From such data, the functional connectivity (FC) between different anatomical nuclei in the brain is constructed, and the functional neuronal networks are determined. Thus, rsfMRI permits to unravel the disturbances of the functional neuronal networks during development of cerebral diseases and their functional improvements after therapeutic interventions.

The study of many cerebral diseases and brain lesions such as e.g., neurodegenerative diseases or stroke and the exploration of effective therapeutic strategies requires long-term monitoring, often of several months (Ramos-Cabrer et al., 2010; Wiesmann et al., 2017; Green et al., 2019). A factor that is, however, often overlooked in rsfMRI studies of mice is the progressing age of the individuals during the required longitudinal studies. Here, we present a rsfMRI study on the effects of aging on FC in the healthy mouse brain in the range of 2–13 months of age. For the analysis of brain connectivity, we have combined Independent Component Analysis (ICA) to denoise rsfMRI data, and Seed-based Correlation Analysis (SCA) to study the correlation between various cortical and subcortical regions of interest (ROIs), while focusing on the sensorimotor networks and the default mode network (DMN). For the first time, we describe changes of the mouse brain connectome during healthy aging, defining an inverse U-shape curve for the FC that peaks at the age of 8–9 months followed by substantial continuous decrease during progressing aging.

## Materials and Methods

### Animals and Experimental Protocol

All animal experiments were performed in accordance with the guidelines of the German Animal Welfare Act and approved by the local authorities (Landesamt für Naturschutz, Umwelt und Verbraucherschutz NRW). *Ad libitum* access to food and water was provided to the animals under a controlled light environment (12 h light/dark).

Twenty-four C57BL/6J male mice (Janvier, Le Genest-St Isle, France) were studied, subdivided into four groups of age: 2 months (*n* = 6), 5 months (*n* = 6), 8 months (*n* = 6) and 12 months (*n* = 6). Each group was scanned twice with a 1 month gap between both acquisitions. Thus, eight time points in total were covered in the study: 2, 3, 5, 6, 8, 9, 12 and 13 months.

### MRI

MRI measurements were carried out on a dedicated animal MRI scanner (Bruker BioSpec, Ettlingen, Germany) operating with a horizontal magnet at 9.4T. Radio frequency (RF) excitation and signal reception were performed with a cryogenic 1H quadrature surface coil (CryoProbe, Bruker BioSpin, Ettlingen, Germany). Monitoring of physiological parameters was achieved with a 1025T System (SA Instruments, Stony Brook, New York, NY, USA) and recorded with DASYlab Software (National Instruments, Austin, TX, USA). Body temperature was measured with a fiber optic rectal probe (SA Instruments, Stony Brook, New York, NY, USA) and kept at 37°C ± 1°C by a water circulating system (Medres, Cologne, Germany). Anesthesia was induced in all mice with isoflurane (3.5%) in air mixture of N_2_ (70%) and O_2_ (30%), and was reduced to 2% isoflurane in the scanner, where the animal’s head was fixed with ear bars and a tooth holder in a dedicated MR compatible animal cradle.

MRI experiments were conducted using Paravision 6.01 (Bruker BioSpin, Ettlingen, Germany). Isoflurane was kept at 1.5%–1.8%, thoroughly adjusted throughout the duration of the experiments, for keeping the breathing rate stable (100–120 bpm). A single bolus of 0.1 mg/kg medetomidine (Domitor, Elanco) was subcutaneously administered suspended in 250 μl of NaCl, 15–20 min before functional imaging acquisition. Within 5 min following the medetomidine injection, isoflurane was decreased to 0.5%–0%, maintaining a maximum of 100–120 breaths per minute during the complete functional imaging data acquisition.

An anatomical reference TurboRARE scan was acquired with the following parameters: TR/TE = 5,500 ms/32.5 ms, matrix = 256 × 256, field of view (FOV) = 17.5 mm × 17.5 mm, 48 consecutive (no gap) slices of 0.3 mm, RARE factor of 8, and 2 averages. Then, an adapted gradient echo-planar imaging protocol (Grandjean et al., 2014) was used for functional image acquisition TR/TE = 2,840 ms/18 ms, FOV = 17.5 mm × 17.5 mm, matrix = 96 × 96, in-plane resolution = 182 μm × 182 μm, 16 slices of 0.5 mm with 0.1 mm inter-slice gap. Once the scanning protocol was completed, a 1 mg/kg Atipamezol (Antisedan, Pfizer), suspended in 100 μl of NaCl, was subcutaneously administered to reverse the effects of medetomidine.

### Data Processing

All datasets were brain extracted using FSL [FMRIB (Oxford Centre for Functional MRI of the Brain) Software Library^1^, (Jenkinson et al., 2002; Smith, 2002)]. Preprocessing of rsfMRI data was performed with single-session Probabilistic ICA (pICA) with the MELODIC interface of FSL^2^, following an adapted procedure of Bajic et al. (2017). This preprocessing consisted of motion correction with MCFLIRT (Jenkinson et al., 2002), high-pass temporal filtering (>0.01 Hz) and registration to the anatomical reference image set (TurboRARE images) which was registered to an in-house mouse brain template. A threshold of *p* < 0.05 was applied to the *z*-scores spatial maps of the independent components provided by MELODIC, before being manually classified into signal or noise, based on information offered by independent component spatial maps, power spectra and time series (Griffanti et al., 2017). In order to achieve the cleaning of data, the components classified as noise were regressed. Following data denoising, a 0.3 mm full-width half maximum (FWHM) Gaussian kernel was applied for spatial smoothing.

Several brain ROIs were selected for FC analysis. Cortical regions include the primary somatosensory cortex (S1), the secondary somatosensory cortex (S2), primary and secondary motor cortex (M1/2), the visual cortex (VC) and the auditory cortex (AC). Subcortical nodes are the caudate putamen (CPu) and the thalamus (Th). Moreover, several regions of the DMN were extracted: the entorhinal cortex (EntC), prelimbic cingulate (Cg), the rostral dorsal prelimbic cortex (PrL), the retrosplenial granular and dysgranular cortex (RSG/RSD), the globus pallidus (GP), the hypothalamus (Hyth) and the hippocampus (Hp). The DMN is regarded as the basal activity network of the brain (Stafford et al., 2014).

Group analysis was conducted by using a customized version of FSLNets (v0.6^3^) in five main steps: (1) averaging of time series in each ROI; (2) calculate full Pearson correlation between pairs of ROIs; (3) transformation of Pearson correlation *r* values to *z*-score by applying Fisher transformation to normalize data; (4) calculate group mean values for each correlation; and (5) build matrices representing *z*-score values between pairs of nodes (i.e., regions).

### Analysis of Age Profile of Functional Connectivity

To construct the age profile of the FC, we followed two strategies. As first approach, we plotted the mean correlation coefficients vs. time for each single measured temporal point (2, 3, 5, 6, 8, 9, 12 and 13 months of age). Alternatively, we plotted the mean correlation coefficients vs. time, averaging the two temporal data sets for each group of animals studied (each group was scanned twice in consecutive months), thus resulting in four temporal points at 2.5 (averaging month 2 and 3 for animal group 1), 5.5 (averaging month 5 and 6 for animal group 2), 8.5 (averaging month 8 and 9 for animal group 3) and 12.5 (averaging month 12 and 13 for animal group 4) months.

### Statistical Analysis

Prism X.0 (Graphpad Software, San Diego, CA, USA) was used for the statistical analyses. D’agostino-Pearson normality test was performed to assess the distribution of data for each network or cross-correlation of interest. In case data was normally distributed for all groups, an analysis of the variance (ANOVA) for repeated measures was performed, followed by a two-tailed unpaired *t*-test. Otherwise, the non-parametric Kruskal–Wallis test was first conducted followed by Mann–Whitney test for pairs of groups. Statistical significances were set at *p < 0.05, **p < 0.01 and ***p < 0.001.

## Results

### Whole-Brain Networks

**Figure 1:**
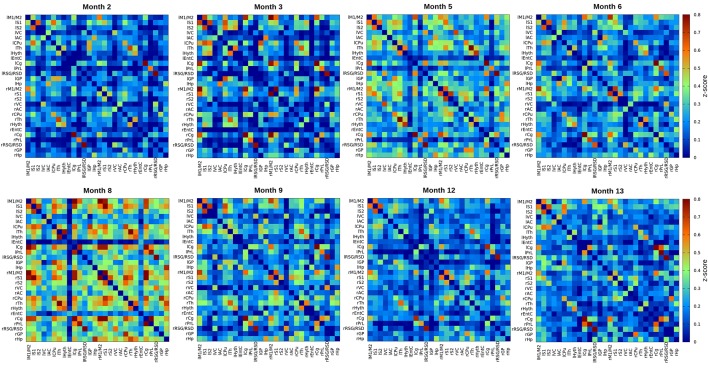
Resting state functional magnetic resonance imaging (rsfMRI) full correlation matrices of the inter- and intra-hemispheric connectivities for 15 regions of interest (ROIs) of the brain. *z*-score values of cross-correlations are plotted. The eight matrices correspond to 2, 3, 5, 6, 8, 9, 12 and 13 months of age of healthy C57BL/6J mice. There is an overall continuous increase of the power of correlation until month 8, indicated by the color change in the LUT. The correlation power decreases again from month 8 until month 13 (*p* < 0.001). The 15 ROIs are: S1, primary somatosensory cortex; S2, secondary somatosensory cortex; M1/2, primary and secondary motor cortex; VC, visual cortex; AC, auditory cortex; CPu, caudate putamen; Th, thalamus; EntC, entorhinal cortex; Cg, prelimbic cingulate; PrL, rostral dorsal prelimbic cortex; RSG/RSD, retrosplenial granular and dysgranular cortex; GP, globus pallidus; Hyth, hypothalamus; Hp, hippocampus. “l” prefix indicates left hemisphere, “r” prefix indicates right hemisphere.

**Figure 2:**
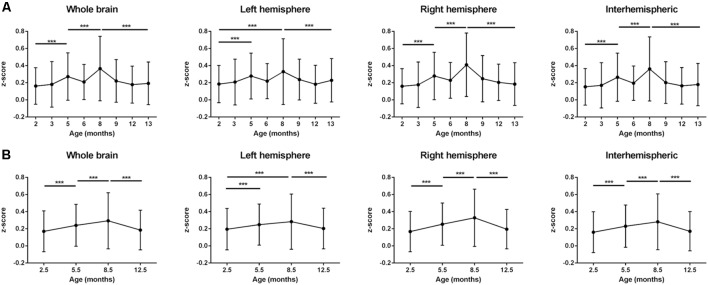
Averaged *z*-score values of left and right intra-hemispheric, inter-hemispheric and whole-brain connectivities. **(A)** *z*-score values, analyzed at the eight experimental time points (2, 3, 5, 6, 8, 9, 12 and 13 months). The power of correlation increases up to month 8 and then decreases again until month 13 in a fluctuating way. **(B)** *z*-scores of equally spaced pairs of time points (averaged *z*-scores for 2–3, 5–6, 8–9, 12–13 months, see “Materials and Methods” section). Smoothed temporal trends show an inverse-U shape curve peaking at month 8.5. Error bars indicate SD. ***p < 0.001.

Color-coded matrices from months 2 to 13, showing the *z*-scores corresponding to interactions between different brain regions, separately analyzed for the left and right hemisphere, are presented in Figure 1. In a first visual inspection, the overall correlation is found to increase from month 2 to month 8 as indicated by the color change in the matrices. After month 8, the connectivity strength decreased again progressively until the last time point at 13 months. Nevertheless, temporal changes are not completely linear and fluctuations of *z*-scores from one time point to the next are considerable when separately analyzing different nodes. To distinguish potential patterns for individual networks, we followed two approaches. First, we decided to group the connectivities in four groups: (1) all intra-hemispheric connectivities of the left hemisphere; (2) all intra-hemispheric connectivities of the right hemisphere; (3) all inter-hemispheric connectivities; and (4) all connectivities within the whole brain (grouping all 2,268 calculated connections together). The averaged *z*-scores of each of these network groups were plotted vs. time (Figure 2A). In addition, we considered that the temporal gap of 1 month between some experimental points is too narrow to attribute changes in correlation to an aging effect, and that variability observed in such short periods could be due to many other experimental or biological factors. Thus, a smoothing of the temporal series was achieved by averaging data with temporal gaps of 1 month (i.e., the pair of scans for each group of animals), resulting in a reduction of the eight measured time points into four evenly spaced time points (3–4 months of temporal resolution, see “Materials and Methods” section. In this way, better interpretable, smoother trends for aging effects were obtained (Figure 2B). As shown in Figure 2B, the mean *z*-score of both, right and left intra-hemispheric groups of correlations, as well as inter-hemispheric and whole-brain connectivities become increasingly stronger from month 2.5 to month 8.5. The increase of the power of correlation from one time point to the next is highly significant for all these periods (*p* < 0.001). After peaking at month 8.5, there is a highly significant decrease in connectivity until the month 12.5 (*p* < 0.001 in all cases).

### Grouping the Whole Brain Into Connectivity Subsets

**Figure 3:**
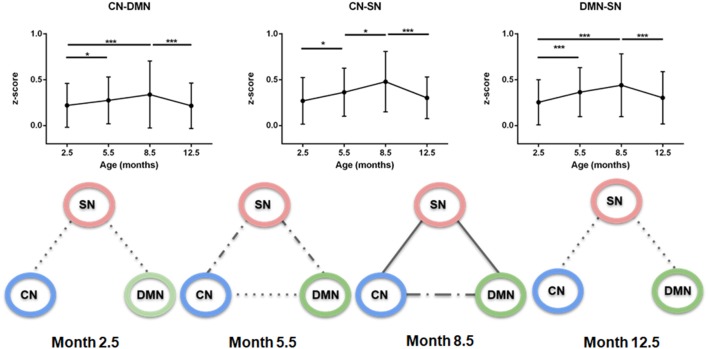
Inter-network connectivities. Upper row: *z*-scores of connections between cortical network (CN), subcortical network (SN) and default mode network (DMN). There is a significant increase of connectivity until 8.5 months, followed by a decrease until month 12.5 in all cases. Lower row: schematic representation of the strength of correlations over time. Each graph, from left to right, represents a specified age (2.5, 5.5, 8.8 and 12.5 months). Solid line, *z*-score > 0.41; Dash-dotted line, *z*-score > 0.33; Dotted line, *z*-score > 0.25; No line, *z*-score < 0.25. CN-SN and SN-DMN connections show a higher correlation over time than the CN-DMN connection (*p* < 0.001). Error bars indicate SD. *p < 0.05 and ***p < 0.001.

In a further analysis, the brain nuclei were grouped in three regions encompassing: (1) the cortical network (CN) consisting of M1/2, S1, S2, AC, and VC; (2) the subcortical network (SN) consisting of CPu and Th; and (3) the DMN (Hyth, EntC, Cg, PrL, RSG/RSD, GP, and Hp). Then, the interactions among these three different networks were studied. The connectivity significantly increased for all three inter-network connections: CN-DMN, CN-SN, DMN-SN networks from month 2.5 to 8.5 (*p* < 0.001 CN-DMN, *p* < 0.001 CN-SN, *p* < 0.001 DMN-SN), as presented in Figure 3. From the age of 8.5 months, a continuous decrease of the inter-network correlations was seen between month 8.5 and month 12.5. Interestingly, the connectivity strength reached at month 8.5 for CN-SN and DMN-SN networks was stronger than that for the CN-DMN (*p* < 0.001 at month 8.5).

### Analysis of Sensorimotor and Default Mode Networks

**Figure 4:**
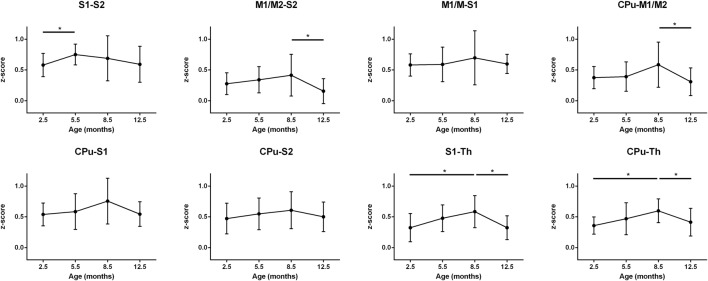
Mean *z*-score values of cross-correlations of pairs of ROIs of the sensorimotor network over the life span period (2.5 months, 5.5 months, 8.5 months and 12.5 months). A significant increase of connectivity was seen from month 2.5 to month 8.5 in S1-Th and CPu-Th connections. The CPu-M1/2, CPu-Th and S1-Th correlations undergo a decrease from month 8.5 to month 12.5. Error bars indicate SD. *p < 0.05.

In the final step, we assessed the individual patterns of connectivities between individual nuclei within the sensorimotor network and the DMN, respectively. A set of the stronger connections within the sensorimotor network is presented in Figure 4. All connectivities share the pattern of the largest *z*-score values at 8.5 months except the interaction between S1 and S2 which reaches its maximal strength already at 5.5 months. This inverse U-shape pattern is most pronounced for the connections M1/2-S2, S1-Th, and CPu-Th. In contrast, the connections M1/2-S1, S1-CPu, S2-CPu have only a very weak, non-significant inverse U-shape during the whole aging process. Interestingly, the *z*-score values at 12.5 months reach low connectivity strength well comparable to those at 2.5 months.

**Figure 5:**
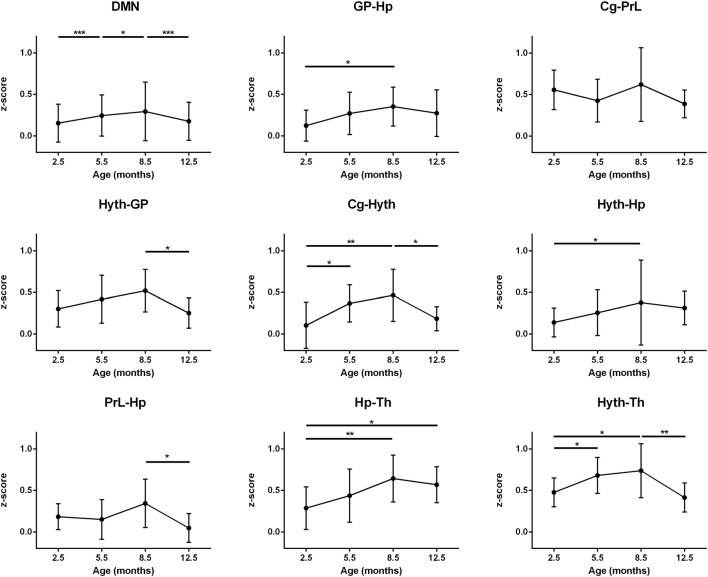
DMN connectivity. Top left: mean *z*-score value of all interactions between DMN regions is represented. All other diagrams: cross-correlation of pairs of ROIs of the DMN is represented. An increasingly strong interaction is seen in month 8.5 comparing to 2.5 in GP-Hp, Cg-Hyth, Hyth-Hp, Hp-Th and Hyth-Th correlations. There is also a decrease of correlation from month 8.5 to month 12.5 in Hyth-GP, Cg-Hyth, PrL-Hp and Hyth-Th. An inverse U-shape curve is typically seen. Error bars indicate SD. *p < 0.05, **p < 0.01 and ***p < 0.001.

Plotting the mean correlation of all interactions of the DMN over time leads to a clear inverse U-shape curve, significantly increasing step-wise from month 2.5 to month 5.5 and month 8.5 followed by a significant decrease to month 12.5 (*p* < 0.001; *p* < 0.05; *p* < 0.001 respectively; Figure 5, top left). Performing an analysis of connections between the individual nodes of the DMN, temporal trends show mostly the same pattern but are more variable than for the sensorimotor network (Figure 5). Most DMN-internal interactions present the strongest correlation at month 8.5, being significantly different from month 2.5 (GP-Hp, *p* < 0.05; Cg-Hyth, *p* < 0.005; Hyth-Hp *p* < 0.05) and show a decline from month 8.5 to month 12.5 (Cg-Hyth, Hyth-GP and the PrL-Hp; *p* < 0.05). A highly significant U-shape pattern of the thalamus (Th) was seen with both Hyth and Hp. Both correlations undergo an increase until month 8.5 (*p* < 0.05), but, while the Hp-Th connection is somehow sustained after month 8.5, the Hyth-Th connection undergoes a considerable decline from that point until month 12.5. The Cg-PrL interaction remained constant over time with a strong connectivity (*z-score* mean = 0.50). Contrary to the sensorimotor network curves, some DMN-internal connectivities do not decrease at 12.5 months to the low *z*-score values at 2.5 months, but stagnate at higher values, although clearly lower than at 8.5 months. This is most pronounced for some connections of the hippocampus: GP-Hp, Hyth-Hp, Hp-Th.

### Condensed Aging Effects of the Functional Networks

**Figure 6:**
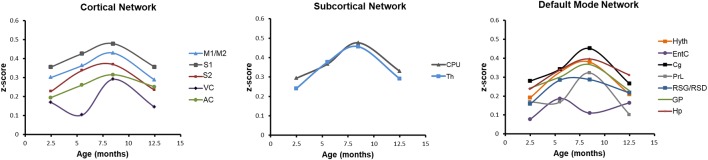
Condensed aging effects of functional connectivity (FC) for individual cortical, subcortical and DMN nodes. Mean *z*-score values of all correlations of each ROI are represented. All nodes show a clear inverse U-shape behavior with a pronounced maximum of the *z*-score values at 8.5 months of age, followed by a substantial drop to low values at 12.5 months of age. Only the entorhinal cortex (right diagram; DMN) shows a deviating behavior with an early maximum at 5.5 months.

Finally, we have studied the average correlation of a selected node with all other nodes across the brain, reflecting the average connectivity strength of this particular node over time. In Figure 6, this behavior pattern is presented for all cortical nodes (Figure 6, left), both subcortical nodes (thalamus and CPu; Figure 6, center) and for all nodes of the DMN (Figure 6, right). In all three groups, the average connectivity strength shows an almost identical U-shape pattern with the maximal values at 8.5 months of age. Only the entorhinal cortex in the DMN group (Figure 6, right) deviates from this pattern and shows an irregular pattern. From these curves, the prominent change in FC strength across progressing aging is clearly seen. Thus, the subcortical FC strength loses 36% of its maximal value at 12–13 months of age in thalamus and CPu. In the cortical node group, the loss of FC strength at 12–13 months varies between 20% for the AC and 50% for the VC. The other cortical nodes have a drop of 33%. In the DMN, the drop of FC is also strongly expressed. While RSG/RSD and Hp show a 24% and 22% drop, respectively, GP, Cg and Hyth experience a much stronger loss of 38% (GP), 40% (Cg), and 45% (Hyth). The strongest effect is noted for the rostral dorsal prelimbic cortex (PrL) with a massive 70% loss in FC strength at 12–13 months of age.

## Discussion

In the present study, we have carefully investigated the age dependence of functional networks by systematic analysis of the rsfMRI data. The age between 2 and 13 months of age was studied, thus covering the most relevant life span phase typically used in chronic long-term experimental mouse models on cerebral diseases and lesions. We have particularly focused on the sensorimotor networks and the DMN as these are most often the relevant functional networks investigated for functional deficits during brain diseases and for functional improvements during therapeutic strategies. Thus, we have unraveled an inverse U-shape behavior of the functional network strength with aging, reaching the maximal strength at 8–9 months of age for both, the sensorimotor networks and the DMN.

The age dependence curve shows similar functional network values shortly after weaning of 2 months of age and at progressed aging at 13 months of age, crossing the maximum strength at 8–9 months. Cortical and subcortical groups presented an overall drop of approximately 33% in network strength when going from 8 to 13 months, with the exception of only a few correlations such as the M1/2-S1 or the Cg-PrL connections which remained rather constant along the whole temporal series. It should be also highlighted that the S1-S2 connection peaked already at month 5.5, unlike the rest of all the studied interactions. In the DMN, the variability in network strength was more pronounced, varying from 24% for RSG/RSD, GP, and Hp to 70% for the rostral dorsal prelimbic cortex (PrL).

Two earlier studies dealing with mouse models of Alzheimer’s disease and focusing on the age dependence of the disease reflected in functional networks changes had also included WT litter mates in their age dependence studies (Grandjean et al., 2014; Shah et al., 2016). Although, the age dependence of the healthy litter mates in those studies was not discussed explicitly in both reports and their focus was primarily on the AD models, information about the age dependence of the rsfMRI data can be derived from their data presentation. Grandjean et al. (2014) had included an age range from 1 to 21 months at variable step sizes, including an age range similar to ours. Careful analysis of the functional networks of the healthy litter mates was limited to the early life phase and data had been recorded in isoflurane anesthesia, different from the present medetomidine-isoflurane mixture, which may affect the functional network results. But from the examples listed in the report of Grandjean et al. (2014), maximum connectivity strength appears to occur between 5 and 8 months of age for the healthy litter mates which agrees well with our present results. In the report by Shah et al. (2016), quantitative analysis of the hippocampus showed a slight increase in connectivity till month 8, and for the prefrontal network a similar increase was reported from 3 to 7 months. Considering the rather low-level information of the age dependence of the healthy litter mates in these two studies, the agreement with our inverse U-shape curve peaking at 8–9 months is very good.

A few recent studies on aging dependence of functional networks in healthy human subjects (Jolles et al., 2011; Bo et al., 2014) point also to a general inverse U-shape of functional network strength. These authors typically compared two or three age groups, defining them as adolescent, adult and aged healthy human subjects where the age span within one group was rather widely defined. These studies confirm our findings in mice that the FC strength increases from early life, reaches a maximum to descend again during progressed aging. Thus, Bo et al. (2014), focusing on the cognitive and motor networks, found an equivalent inverse U-shape behavior for both networks, peaking at the young adult group, aged 18–33 years in their investigation.

Our study shows how FC increases continuously from the second month of life until month 8–9 in the life span of mice, from where a continuous decrease in FC takes place until month 12.5. Although the strength of the FC is variable, the inverse-U shape is robust throughout the whole brain. Underlying reasons for this age-dependent behavior of the functional networks are not understood but a relationship with structural network changes has been considered. During the whole life span, a change of the integrity of white and gray matter content has been reported. Thus, in a study focusing on the structural brain development of young mice, we recently reported a continuous myelination increase in healthy mouse brain up to 6 months of age with parallel cortical thinning, clearly indicating an ongoing morphological change during this period of mouse brain adolescence (Hammelrath et al., 2016). In a study on structural brain networks with 484 healthy subjects aging between 5 and 85 years old, Douaud et al. (2014) described an inverse U-shape pattern. In their study, they revealed how certain regions of the brain thrive in a late stage of adolescence till structural connectivity peaked at approximately 40 years of age, followed by a decrease during aging. With the combined protocol of diffusion spectrum imaging (DSI) and rsfMRI, Green et al. (2019) could show dramatic functional changes without structural changes in mouse models of tauopathy. The same authors moreover reported that the parallel decreases of structural and functional networks strength after stroke were decoupled when a stem cell treatment to the stroke was included in the experimental protocol (Green et al., 2018). Thus, it will be of particular interest in future studies to co-register structural and functional networks using a combined protocol of DSI and rsfMRI to unravel whether structural and functional network changes develop in parallel with healthy aging or whether they may also become decoupled at a certain point. Parallel to structural alterations, hemodynamic parameters may also contribute to the observed age dependence decrease of FC. Thus, Balbi et al. (2015) studied the age-dependent changes of microcirculation in mouse brain. Their most important finding was that neurovascular coupling becomes impaired after 8 months already—where also the maximal FC, observed by us, started to decline—while no change of cellular composition of the neurovascular coupling or impaired Ca^2+^ reactivity was found.

We believe that our studies have an important consequence for the design, performance and analysis of future longitudinal rsfMRI studies in mice. As we have seen, there is a strong effect of aging in mouse connectome, even for temporal periods as short as a few months. Establishing 8–9 months as the age at which connectivity starts to decline and taking into account the progression with age, will benefit to avoid confounds of aging effects underlying the particular aspects of disease-caused functional alterations of slow, long-term functional regeneration processes. In particular, it is between the 8th and 12th month of life where the mouse begins to show signs of deterioration. The first senescent changes take place presumably between 10 and 15 months of age, at 15 months approximately the mouse loses its fertility, and at 18 months aging biomarkers are considered evident (Flurkey et al., 2007). On the other hand, during the early life span till 8–9 months of age, a continuously increasing strength of the functional networks may partly cover decreases caused by cerebral diseases and may lead to overestimation of therapeutic effects during apparent “recovery” of functional networks. Thus, we have planned to extend the study on stroke induction at variable age in mice to clarify stroke-induced FC alterations as a function of age and to decipher whether functional network derangements after stroke are more severe in aged or young mice.

## Summary

FC strength of the sensorimotor and DMNs in the mouse increases from month 2, continuously reaching the maximum at 8–9 months of age. The decrease of the functional network strength after 8–9 months reflects the progressing aging and reaches low values at 12–13 months of age equivalent to those in the early adolescent phase at 2–3 months. In summary, the functional network strength follows a clear inverse U-shape curve during adolescence to maximum at adulthood and progressed aging.

## Data Availability Statement

The raw data supporting the conclusions of this manuscript will be made available by the authors, without undue reservation, to any qualified researcher.

## Ethics Statement

All animal experiments were performed in accordance with the guidelines of the German Animal Welfare Act and approved by the local authorities (Landesamt für Naturschutz, Umwelt und Verbraucherschutz NRW).

## Author Contributions

AE recorded the rsfMRI data, analyzed the data and wrote the manuscript. AM recorded the rsfMRI data and analyzed the data. MD analyzed the data. DW and MH designed the study, analyzed the data and wrote the manuscript. PR-C designed the study and wrote the manuscript.

## Conflict of Interest

The authors declare that the research was conducted in the absence of any commercial or financial relationships that could be construed as a potential conflict of interest. The reviewer SG declared a past co-authorship with one of the authors MH, to the handling Editor.

## Footnotes

^1^http://www.fmrib.ox.ac.uk/fsl

^2^http://fsl.fmrib.ox.ac.uk/fsl/fslwiki/MELODIC

^3^www.fmrib.ox.ac.uk/fsl

## References

[B1] Antony P. M. Diederich N. J. Balling R. (2011). Parkinson’s disease mouse models in translational research. Mamm. Genome 22, 401–419. 10.1007/s00335-011-9330-x 21559878 PMC3151483

[B2] Bajic D. Craig M. M. Mongerson C. R. L. Borsook D. Becerra L. (2017). Identifying rodent resting-state brain networks with independent component analysis. Front. Neurosci. 11:685. 10.3389/fnins.2017.00685 29311770 PMC5733053

[B3] Balbi M. Ghosh M. Longden T. A. Jativa Vega M. Gesierich B. Hellal F. (2015). Dyfunction of mouse cerebralarteries during early aging. J. Cereb. Blood Flow Metab. 35, 1445–1453. 10.1038/jcbfm.2015.107 26058694 PMC4640303

[B4] Binnewijzend M. A. Schoonheim M. M. Sanz-Arigita E. Wink A. M. van der Flier W. M. Tolboom N.. (2012). Resting-state fMRI changes in Alzheimer’s disease and mild cognitive impairment. Neurobiol. Aging 33, 2018–2028. 10.1016/j.neurobiolaging.2011.07.003 21862179

[B5] Biswal B. Yetkin F. Z. Haughton V. M. Hyde J. S. (1995). Functional connectivity in the motor cortex of resting human brain using echo-planar MRI. Magn. Reson. Med. 34, 537–541. 10.1002/mrm.1910340409 8524021

[B6] Bo J. Lee C. M. Kwak Y. Peltier S. J. Bernanrd J. A. Buschkuehl M.. (2014). Lifespan differences in cortico-striatal resting state connectivity. Brain Connect. 4, 166–180. 10.1089/brain.2013.0155 24575740 PMC3994992

[B7] Busche M. A. Eichhoff G. Adelsberger H. Abramowski D. Wiederhold K. H. Haass C.. (2008). Clusters of hyperactive neurons near amyloid plaques in a mouse model of Alzheimer’s disease. Science 321, 1686–1689. 10.1126/science.1162844 18802001

[B8] Douaud G. Groves A. R. Tamnes C. K. Westlye L. T. Duff E. P. Engvig A.. (2014). A common brain network links development, aging, and vulnerability to disease. Proc. Natl. Acad. Sci. U S A 111, 17648–17653. 10.1073/pnas.1410378111 25422429 PMC4267352

[B9] Flurkey K. M. Currer J. Harrison D. E. (2007). “Chapter 20 - mouse models in aging research,” in The Mouse in Biomedical Research (2nd Edition), eds Fox J. G. Davisson M. T. Quimby F. W. Barthold S. W. Newcomer C. E. Smith A. L. (Burlington, VT: Academic Press), 637–672.

[B10] Grandjean J. Schroeter A. Batata I. Rudin M. (2014). Optimization of anesthesia protocol for resting-state fMRI in mice based on differential effects of anesthetics on functional connectivity patterns. Neuroimage 102, 838–847. 10.1016/j.neuroimage.2014.08.043 25175535

[B11] Green C. Minassian A. Vogel S. Diedenhofen M. Beyrau A. Wiedermann D.. (2018). Sensorimotor functional and structural networks after intracerebral stem cell grafts in the ischemic mouse brain. J. Neurosci. 38, 1648–1661. 10.1523/jneurosci.2715-17.2018 29321138 PMC6705873

[B12] Green C. Sydow A. Vogel S. Anglada-Huguet M. Wiedermann D. Mandelkow E.. (2019). Functional networks are impaired by elevated tau-protein but reversible in a regulatable Alzheimer’s disease mouse model. Mol. Neurodegener. 14:13. 10.1186/s13024-019-0316-6 30917861 PMC6438042

[B13] Griffanti L. Douaud G. Bijsterbosch J. Evangelisti S. Alfaro-Almagro F. Glasser M. F.. (2017). Hand classification of fMRI ICA noise components. Neuroimage 154, 188–205. 10.1016/j.neuroimage.2016.12.036 27989777 PMC5489418

[B14] Hammelrath L. Škokićc S. Khmelinskii A. Hess A. van der Knaap N. Staring M.. (2016). Morphological maturation of the mouse brain: an *in vivo* MRI and histology investigation. Neuroimage 125, 144–152. 10.1016/j.neuroimage.2015.10.009 26458518

[B15] Huang G. Ashton C. Kumbhani D. S. Ying Q. L. (2011). Genetic manipulations in the rat: progress and prospects. Curr. Opin. Nephrol. Hypertens. 20, 391–399. 10.1097/mnh.0b013e328347768a 21546835 PMC3857098

[B16] Jenkinson M. Bannister P. Brady M. Smith S. (2002). Improved optimization for the robust and accurate linear registration and motion correction of brain images. Neuroimage 17, 825–841. 10.1016/s1053-8119(02)91132-8 12377157

[B17] Jolles D. D. van Buchem M. A. Crone E. A. Rombouts S. A. (2011). A comprehensive study of whole-brain functional connectivity in children and young adults. Cereb. Cortex 21, 385–391. 10.1093/cercor/bhq104 20542991

[B18] Lutz C. (2018). Mouse models of ALS: past, present and future. Brain Res. 1693, 1–10. 10.1016/j.brainres.2018.03.024 29577886

[B19] Ramos-Cabrer P. Justicia C. Wiedermann D. Hoehn M. (2010). Stem cell mediation of functional recovery after stroke in the rat. PLoS One 5:e12779. 10.1371/journal.pone.0012779 20877642 PMC2943902

[B20] Ransohoff R. M. (2012). Animal models of multiple sclerosis: the good, the bad and the bottom line. Nat. Neurosci. 15, 1074–1077. 10.1038/nn.3168 22837037 PMC7097342

[B21] Shah D. Praet J. Latif Hernandez A. Höfling C. Anckaerts C. Bard F.. (2016). Early pathologic amyloid induces hypersynchrony of BOLD resting-state networks in transgenic mice and provides an early therapeutic window before amyloid plaque deposition. Alzheimers Dement. 12, 964–976. 10.1016/j.jalz.2016.03.010 27107518

[B22] Smith S. M. (2002). Fast robust automated brain extraction. Hum. Brain Mapp. 17, 143–155. 10.1002/hbm.10062 12391568 PMC6871816

[B23] Stafford J. M. Jarrett B. R. Miranda-Dominguez O. Mills B. D. Cain N. Mihalas S.. (2014). Large-scale topology and the default mode network in the mouse connectome. Proc. Natl. Acad. Sci. U S A 111, 18745–18750. 10.1073/pnas.1404346111 25512496 PMC4284535

[B24] Wiesmann M. Zinnhardt B. Reinhardt D. Eligehausen S. Wachsmuth L. Herrmann S.. (2017). A specific dietary intervention to restore brain structure and function after ischemic stroke. Theranostics 7, 493–512. 10.7150/thno.17559 28255345 PMC5327363

